# Scanning acoustic microscopy for material evaluation

**DOI:** 10.1186/s42649-020-00045-4

**Published:** 2020-11-05

**Authors:** Hyunung Yu

**Affiliations:** Korea Research Institute of Science and Standards, Daejeon, 34113 South Korea

**Keywords:** Microscopy, Scanning, Acoustic, Defect, Delamination, Crack, Void, Non-destructive, Analysis

## Abstract

Scanning acoustic microscopy (SAM) or Acoustic Micro Imaging (AMI) is a powerful, non-destructive technique that can detect hidden defects in elastic and biological samples as well as non-transparent hard materials. By monitoring the internal features of a sample in three-dimensional integration, this technique can efficiently find physical defects such as cracks, voids, and delamination with high sensitivity. In recent years, advanced techniques such as ultrasound impedance microscopy, ultrasound speed microscopy, and scanning acoustic gigahertz microscopy have been developed for applications in industries and in the medical field to provide additional information on the internal stress, viscoelastic, and anisotropic, or nonlinear properties. X-ray, magnetic resonance, and infrared techniques are the other competitive and widely used methods. However, they have their own advantages and limitations owing to their inherent properties such as different light sources and sensors.

This paper provides an overview of the principle of SAM and presents a few results to demonstrate the applications of modern acoustic imaging technology. A variety of inspection modes, such as vertical, horizontal, and diagonal cross-sections have been presented by employing the focus pathway and image reconstruction algorithm. Images have been reconstructed from the reflected echoes resulting from the change in the acoustic impedance at the interface of the material layers or defects. The results described in this paper indicate that the novel acoustic technology can expand the scope of SAM as a versatile diagnostic tool requiring less time and having a high efficiency.

## Introduction

Ultrasound refers to a sound wave having a frequency (> 20 kHz) higher than the values a human being can hear. Wild animals such as bats and dolphins use acoustic waves to detect and locate objects and obstacles and catch prey in a dark environment. Acoustics are not only utilized in military, scientific, and industrial fields, and medical imaging but also in new and efficient evaluation techniques for quantitatively characterizaing the physical properties such as strain, distance, and viscoelasticity of elastic and hard materials (Maev 2008; Maev 2013; Maev 2016; Saijo 2009; Schubert et al. 2018).

Interest in acoustics began very early in the sixth century BC when Pythagoras’ proposed the mathematical properties of the strings on instruments. The application of acoustic microscopy was initiated by Sokolov in 1949 and a breakthrough was achieved in the early 1970s in high-resolution imaging for investigating the internal structure of nontransparent solids (Maev 2016; Bertocci et al. 2019). Acoustic microscopy has been substantially improved by Quate at Stanford University in 1985 for achieving an accurate damage or defect detection in microelectronics and early diagnosis of mutations in the tissues or organs as a non-invasive analytical method (Saijo et al. 1997; Bilen et al., 2019a, b; Kubit et al., 2019).

Optical microscopy, X-ray computer-aided tomography, infrared thermography, and scanning acoustic microscopy (SAM) are the commonly utilized techniques owing to their short time consumption and low sample pretreatment requirement as compared to electron microscopy (Sheppard, 2020; Bertocci et al. 2019). The most striking advantage of SAM is its ability to inspect the sample subsurfaces layer by layer simultaneously with an excellent penetrating power of the ultrasonic waves while scanning the material surface (Morokov and Levin, 2019; Brand et al. 2014). Thus, three-dimensional (3D) tomography is possible for inner structure integration by using complex and sophisticated algorithms of signal processing with a lateral two-dimensional scan (Maev 2008; Maev 2016; Saijo 2009). At present, the ultrasonic technique has become a robust method for non-invasive material evaluation and defect detection for conducting a quality assessment and metrology in a short time and with high efficiency (Kustov and Miguel, 2019; Bilen et al., 2019a, b; Kim et al. 2019; Zhu et al. 2020).

In this paper, the basic principles and applications of SAMs are described with a collection of results and techniques.

## Basic principles of SAM

A SAM consists of an ultrasonic emitting transducer, a mechanical scanner and an image processor. The transducer plays an important role as a lens that delivers and focuses the acoustic wave generated by the piezoelectric array and as a detector that accepts the echo reflected from the sample. The ultrasonic pulses (acoustic waves) are generated by a piezoelectric transducer comprising of a magnet and a radiofrequency coil. The transducer crystals are made up of lithium niobate (LiNbO_3_) single crystals, lead magnesium niobate-lead titanate (PMN-PT) single crystals, quartz or piezoelectric ceramics for frequencies below 100 MHz. Above this limit, a zinc oxide (ZnO) crystal is often used. The piezoelectric transducer comprises a lens, a matching layer, and active piezoelectric array elements that are connected to electrical lines. The acoustic signal oscillates at its own frequency when it receives an electronic intermittent pulse excitation and is delivered via a sapphire cylinder (Al_2_O_3_) to the lens. The flat wavefront focuses along the z-axis after refraction at the lens/coupling medium interface. Refraction occurs at the interface because of the different velocities of the sound waves within the two materials. Since most materials have a higher speed than a water coupling medium, the focal length is shortened. This phenomenon can be caused by refraction. Acoustic coupling via a water medium between the lens (transducer) and the sample (object) is very important for an efficient delivery of the acoustic waves.

Variations in the acoustic impedance at the interfaces of the tested material lead to the reflection and scattering of the ultrasonic waves. A signal is acquired after the interaction of the acoustic pulse with the sample in the reverse order. The reflected signal (echo) is collected with a diverging form, but is transformed into a flat wave after a lens and is then converted into an electrical signal with a spatial coordinate. The electrical voltage is amplified and digitized using a image processor.

Using a raster scanning motion of the transducer on the sample surface, the acoustic echo signals are collected at each point and the depth information is recorded in a histogram. 3D images can be reconstructed from all recorded histograms for the region of interest. Various scanning pathways are possible for handling the algorithms of the transducer. More details of the scan motions are explained later. A time duration must be recorded when a series of acoustic excitations occurs owing to the transit time (*t*) of the reflections or echoes in the sample. The repetition rate is limited by a processor-controlled process depending on the frequency used. The lower the frequency, the longer are the pulses, but the attenuation decreases.

### See-through inspection using SAM

**Scheme 1:**
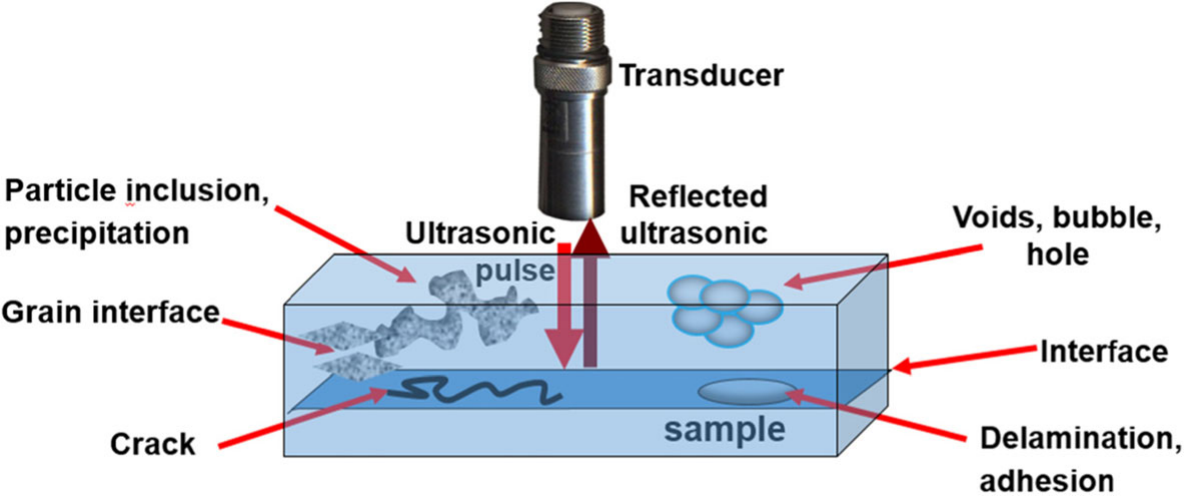
Schematic showing the probing pulse and the reflected echo detected from the internal structure of a sample consisting of a variety of defects and inclusions.

Scheme 1 shows the schematic of a reflection mode SAM applied to a material that includes various defects such as grain boundaries, cracks, delamination, voids, bubbles, and contaminants. The SAM uses an ultrasonic pulse from the transducer to probe the sample and monitors the reflected echoes from a series of time delays of the pulses induced after interacting with the internal structure. These delays depend on the acoustic properties of the material, such as its density and attenuation that is determined by the acoustic impedance of the material.

**Fig. 1:**
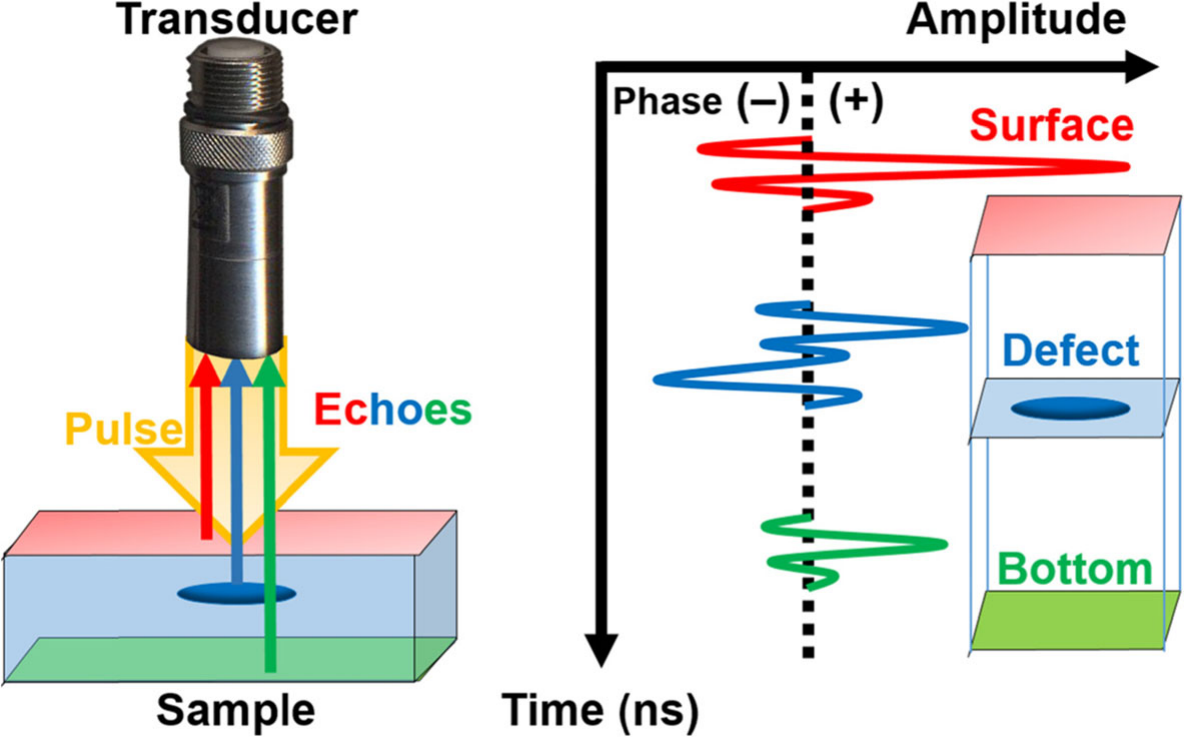
Schematic showing the incident and reflected ultrasonic pulse (left) and histogram (right) showing the amplitude of the reflected echo (A-scan) after an ultrasonic pulse interacts with a sample

The ultrasonic pulse generated from the transducer is delivered to the sample via a water medium. During this process, some fraction of the pulse bounces off the surface owing to the change in the acoustic impedance (Fig. 1). The ratio of the reflected waves to the transmitted waves is determined from the impedance mismatch. The acoustic impedance (Z) is defined as the amount of resistance an ultrasound beam experiences as it passes through a material and is expressed as follows:

1 $$ \mathrm{Z}=\uprho \times \mathrm{c} $$

where ρ material density (in kg/m^3^), c is the velocity of sound within the material (in m/s) and Z is related to the acoustic hardness of the material. Therefore, if the density of the material increases, its impedance increases. Similarly, when the velocity of sound in the material increases, its impedance also increases. When an ultrasound beam encounters an interface at normal incidence, some of the energy is reflected (reflection) off its surface and some amount is transmitted (transmission) through the interface. The amplitude of the reflected echo depends on the acoustic impedance at the interface or the boundary between the two materials. We can intuitively understand that most of the sound is reflected when the difference in acoustic impedance is large. The ultrasound intensity or the amount of the reflected and transmitted waves can be evaluated using the reflection coefficient, R, and the transmission coefficient, T, respectively as expressed by (Dukhin and Goetz, 2010),

2 $$ R=\frac{I_R}{I_0}=\frac{{\left({Z}_2-{Z}_1\right)}^2}{{\left({Z}_2+{Z}_1\right)}^2} $$

3 $$ T=\frac{I_T}{I_0}=1-R=\frac{4{Z}_2{Z}_1}{{\left({Z}_2+{Z}_1\right)}^2} $$

Where Z_1_ and Z_2_ are the acoustic impedances of 1st and 2nd material.

For example, the amount of energy or intensity that is reflected at the water-to-polypropylene interface, using the acoustic impedance values of water and polypropylene of 1.48 and 2.48, respectively, becomes R = 0.0638. Thus, 6.38% intensity is reflected and 93.6% is transmitted.

The higher the acoustic impedance mismatch at the interface, the larger is the intensity of the reflection or brighter is the reflected image. The energy loss is represented as an attenuation while the ultrasound beam progresses through a medium. In practice, we should also consider three major attenuation factors other than reflection and transmission, namely, diffraction, scattering, and absorption of the ultrasound beam.

An acoustic pulse passes through three boundaries, namely, the surface, defects, and the bottom, and the time-of-flight (t) of the pulse reflected from each surface provides the depth information of the sample. A series of reflected waveforms are recorded in real time with a phase histogram, called, the A-scan of the reflection mode SAM. The ultrasonic wave experiences a phase inversion at the interface of the high and low acoustic impedance region, thus resulting in a minimum of three consecutive amplitudes of the signal. The (+)and (−) phase signals should cross the baseline (vertical dotted line in Fig. 1). The SAM instrument automatically detects a histogram with a phase often caused by irregularities inside the sample such as delamination, void, or a hole. The ultrasonic wave propagation in the acoustic microscope results in the wave front snapshots calculated along the time-of-flight of the echo.

**Fig. 2:**
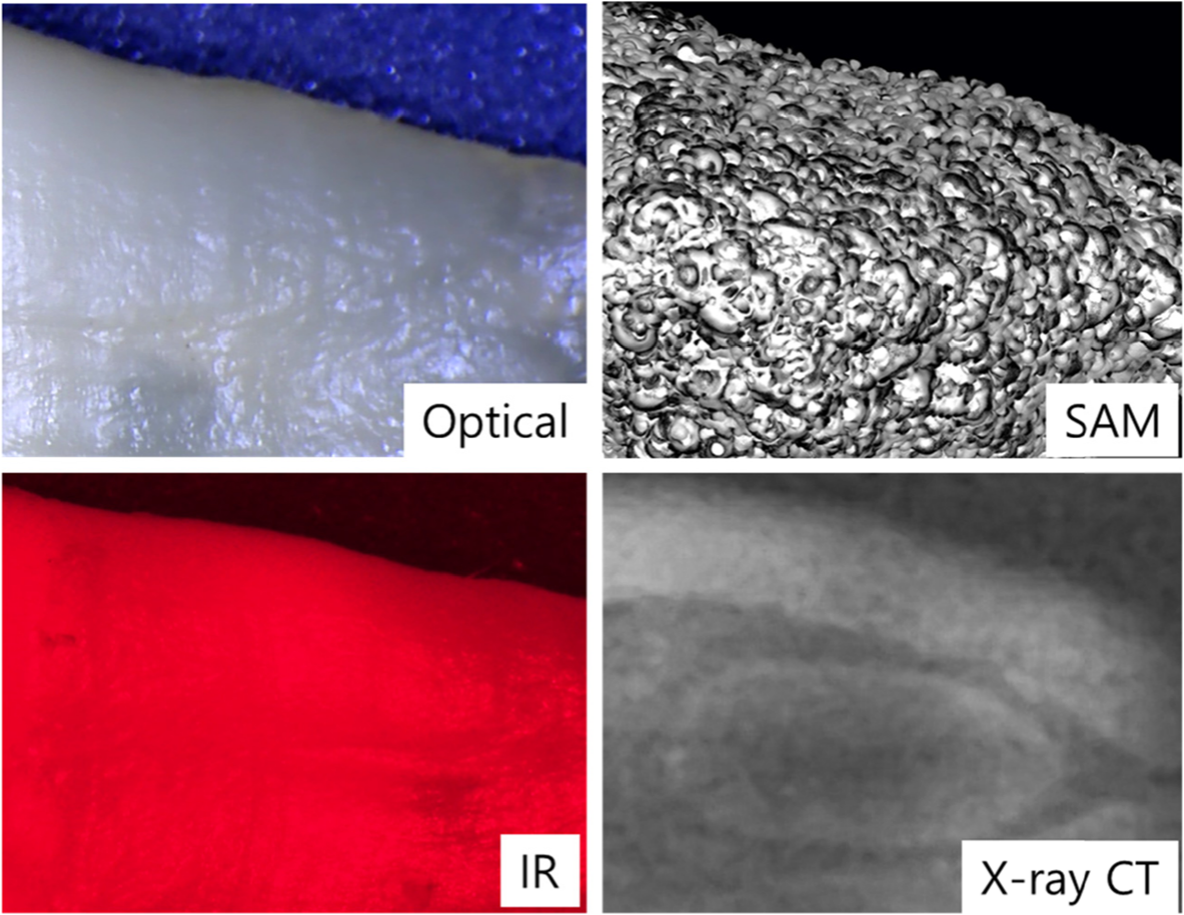
Comparison of the analysis of the interior of the tooth using different imaging techniques.

Figure 2 shows the results of the analysis conducted of the tooth using different analytical imaging instruments. Optical microscopy is the most frequently used methods for easy and fast visual inspection, but is often limited to surface investigations. Infrared imaging is useful for functional group analysis of the chemical bonding, contamination or defects. However, it provides a very low image resolution because of the limited range of infrared wavelengths, is costly, has a low sensitivity of the camera, and it is sometimes difficult to use this technique in a moist atmosphere. X-ray computed tomography (CT) is good for transmission imaging but is not highly effective for imaging materials having a high Pb content or materials having small density differences, for example, adhesion or defect analysis between high-density materials such as a metal-metal bonding (Bertocci et al. 2019) is difficult. The SAM image shows dentin clear domain structures of underlayer surface.

### The SAM analysis modes

**Fig. 3:**
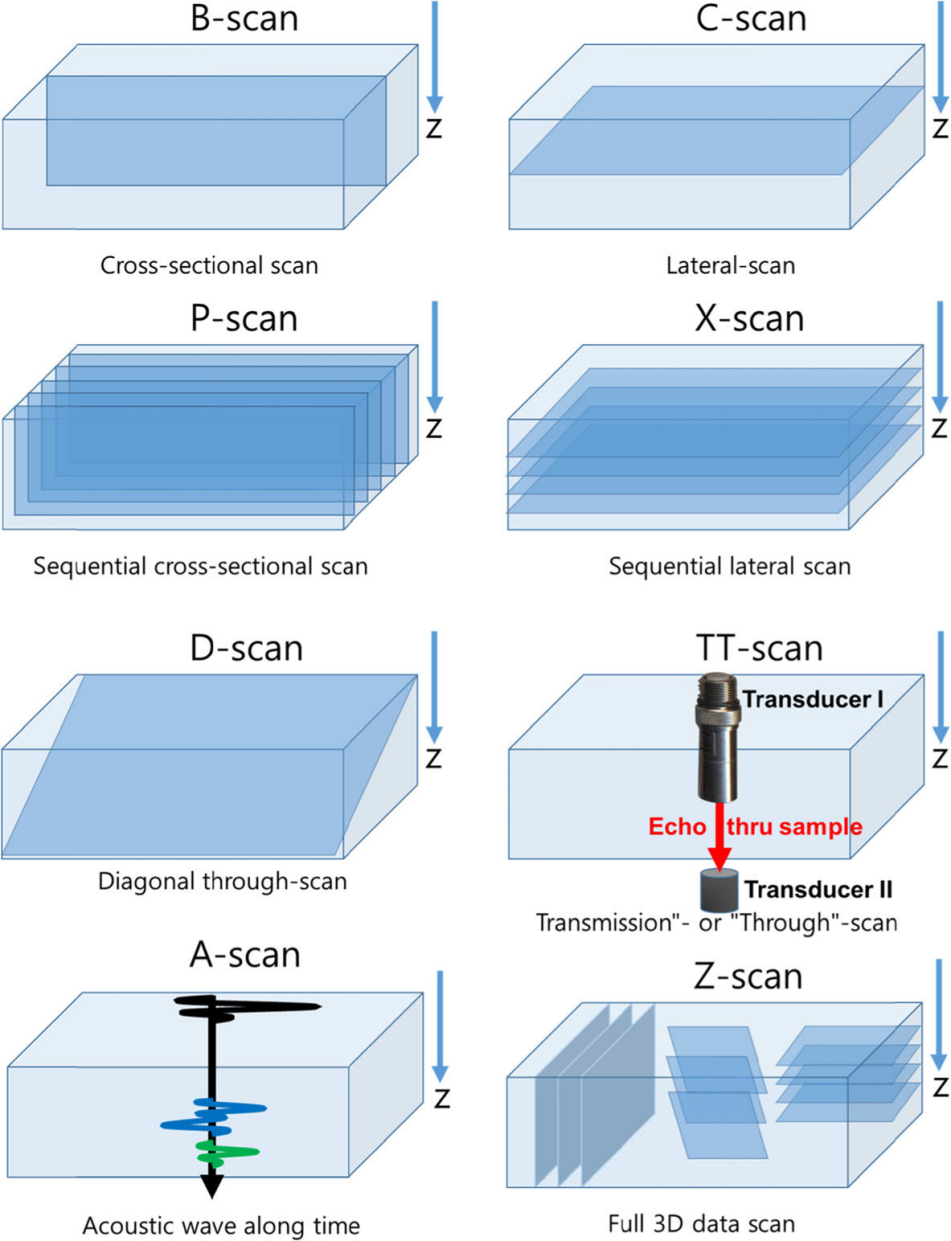
Different types of scanning analytical modes of SAM

Figure 3 shows the different types of scanning analytical modes of SAM used for material characterization. The basic three modes are the A-scan, B-scan, and C-scan. An A-scan is a raw waveform which records the echo amplitude along the ultrasonic transit time. In this mode, the optimal conditions are controlled by tuning the amplitude at the maximum so that the transducer is focused on a point of interest. After the scanning in the A-scan mode is completed, acoustic images can be further processed by a B-scan or a C-scan. The B-scan represents a cross sectional view through the material that provides the depth information, whereas a C-scan records a single image of the selected layer at a specific depth. In either scan modes, it is important to move the transducer along each vertical axis until the echo signal is reflected at a moderate intensity for the region of interest.

When a rough or tilted surface is scanned, a sufficient trigger level and trigger width must be set for activating the receiving trigger signal over the whole surface. For further functions of the scan, a series of images at equidistances is possible without any difficulty using a P-scan (cross-sectional) and an X-scan (multi co-planar) mode, respectively.

In particular, the X scan is highly recommended for an unknown sample to obtain an overview of the sample structure after an A-scan. *The number of image stacks depends on the B scan gate width divided by the width of the data gate (depth resolution).* The D scan is a diagonal scan through the sample. The Z-scan is a useful integration of several modes, but requires a very long time. The data obtained with Z-scan can be conveniently loaded at a later time for detailed sample examination. The transmission through scan (TT-scan) looks through the entire sample in one single scan, and thus all defects are visible in a single panfocal image (Takezaki et al. 2019). In the TT-scan mode, the transmitted signal is acquired with the 2nd transducer instead of the reflected echo which is appropriate for a thin material.

### Resolution and penetration depth

Similar to the optical microscope, the main parameters of SAM are the resolution, penetration depth, and contrast. The resolution, w, of a microscope used for imaging is determined by the ultrasonic frequency, *f*, used

4 $$ w=\frac{\uplambda}{2\ \mathrm{NA}} $$

5 $$ f\times \lambda =\mathrm{c} $$

where λ is the wavelength of the ultrasound wave in the liquid, NA is the numerical aperture,

and c is the velocity of sound in the material.

Substituting λ from Eq. (5) into Eq. (4), we get

6 $$ w=\frac{\mathrm{c}}{2f\times \mathrm{NA}} $$

Equation (6) indicates that the imaging resolution can be reduced by increasing the frequency.

Since the aberrations in the SAM are negligible, its resolution is determined by the wavelength used according to the diffraction limit, where

7 $$ w=\frac{\uplambda}{2\ \mathrm{NA}} $$

NA is the numerical aperture of the acoustic lens. NA = sin θ, where θ is the semi-angle of the lens aperture subtended at the focus. A well-designed lens can obtain a focal spot at 0.7 μm using a transducer of 2 GHz frequency in water but the penetration depth is limited to only a few microns for surface investigations, such as probing a tooth enamel or examination of carotid arterial plaques (Saijo et al. 2002). The advantage of SAM is that enables the visualization of the material structure below the surface of a non-transparent solid owing to favorable propagation property of acoustic waves. The penetration depth can be estimated primarily using the elastic parameters of the sample, the operating frequency of the acoustic lens, and the signal-to-noise ratio. By optimizing the opening angle of the lens, a satisfactory coupling between the sample and the liquid can be obtained and this will increase the penetration depth of the incident ultrasound beam (Liang et al. 2019; Song et al. 2013). The higher the acoustic mismatch due to the impedance difference between the object and the liquid, the lower is the penetration depth. For instance, compared to the high-impedance materials, the low-impedance objects shear and the longitudinal waves can reach considerably into the interior while imaging. In addition, a lower frequency results in a deeper penetration because of its lower attenuation with frequency. Most available frequency ranges between 10 and 150 MHz are optimized for subsurface imaging analysis.

An A-scan shows the amplitude along the time, t, which provides the depth, d, of a defect in the material according to the following relation (Liang et al. 2019; Song et al. 2013):

8 $$ d=\frac{t\times c}{2} $$

Here, c represents the velocity of sound within the material and the factor 2 accounts for the round trip of the sound wave. For example, the depth in water at 67.89 μs is 50.00 mm while that in epoxy resin is 88.26 mm, as follows.

9 $$ 50.00\  mm=\frac{1.473\  mm/\mu s\times 67.89\ \mu s}{2} $$

10 $$ 88.26\  mm=\frac{2.600\  mm/\mu s\times 67.89\ \mu s}{2} $$

A variety of imaging instruments are available for surface and interface analyses. Compared to the electron microscope, non-invasive imaging instruments are less time-consuming in terms of sample preparation and are beneficial for sample recycling depending on the damage done during measurement exposure (Table 1). A scanning acoustic microscope is one of the useful and a highly efficient method of quality and safe assessment of sensitive and expensive products (Kustov and Miguel, 2019; Bilen et al., 2019a, b; Kim et al. 2019; Zhu et al. 2020).

**Table 1:** Imaging methodology for interface analysis^a^

Technology	Highest lateral resolution	Highest depth resolution	Scanning time
Infrared	250 μm	250 μm	3 min
X-ray tomography	10 μm	> 2 μm	30 min
Scanning Acoustic Microscope	< 1 μm	1 μm	2–8 min
Optical Microscope	0.3 μm	1 μm	5 min
Transmission Electron Microscope	0.1 nm	50 nm	60 min

^a^some data courtesy of PVA TePla Analytical System

The measured focal length of a transducer depends on the material on which it is being measured, since different materials have different sound velocities. As the sound velocity in most materials is higher than in water, the focal length of the transducer is effectively shortened when specified with water and this effect is caused by refraction. The resolution and penetration depth of the acoustic microscope are summarized in Table 2 to show a trade-off behavior of the improved resolution at the expense of the penetration depth with increasing transducer frequency. Ultrasound waves of low frequencies such as 5 MHz, penetrate up to 15.0 mm deeper into the materials than the ultrasound of higher frequencies, but the spatial resolution of the acoustic image decreases. In contrast, ultrasound waves of very high frequencies lead to a very high resolution; however, they do not penetrate deeply.

**Table 2:** Resolution and penetration depth of an acoustic microscope^a^

Frequency (MHz)	Penetration depth (mm)	Theoretical lateral resolution (μm)	Focal length (mm)
5	15.0	300	19.0
10	10.0	150	15.0
15	5.1	100	19.0
20	4.1	75	15.0
25	4.1	60	15.0
30	3.4	50	12.7
40	5.4	38	20.0
75	3.4	20	12.7
80	2.2	19	8.0
100	0.4	15	1.5
110	2.2	14	8.0
120	2.2	12	8.0
200	0.3	8	7
1000	0.025	1.5	0.08
2000	0.010	0.7	0.05

^a^some data courtesy of Kramer Scientific Instruments System

## Experimental details

In the experiment conducted in this study, a sample was placed in a pure water tank of the SAM (KSI V8, Kramer Scientific Instruments, Herborn, Germany). A transducer was mounted on the z-axis and could be moved above the sample center. Unlike the case of the optical microscope, the air becomes a poor transmitter of acoustic waves from the transducer in the SAM up to the sample. Thus, the sample was completely immersed in deionized, degassed water approximately 15 mm below the transducer depending on its focal length (Bertocci et al. 2019). The sample was fixed tightly to avoid any disturbance to it by the wave turbulence while performing the scan using the transducer. In addition, the transducer was positioned to be coplanar to the sample as much as possible so that the reflection of the echo signal would be maximum and would provide the best image contrast.

In addition to the abovementhioned points, it was also important to remove the tiny air bubbles under the transducer lens using a soft brush after dipping the transducer into the water or after the degassing procedure. The transducer was then moved along the Z-direction, i.e. along the depth close to the sample, until the amplitude of the A-scan reached its maximum at a proper working distance of the transducer.

## Results of the SAM inspection of hard and elastic materials

**Fig. 4:**
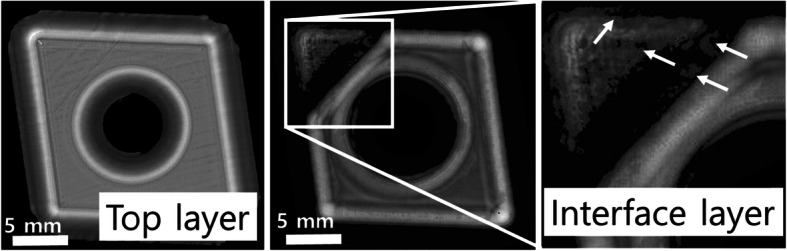
Images of tungsten carbide inserts obtained from a C-scan using SAM. Incomplete adhesion is indicated by arrows in the image of the interface layer (right) (110 MHz transducer, lateral resolution ~ 15 μm, 512 px × 512 px)

Figure 4 demonstrates the adhesive composite structure between tungsten carbide and the shank with variations in the adhesive layer thickness. The composite bites bonded with epoxy are widely used in high performance cutting parts of automatic lathes and micromechanics (Yared et al. 2019). The performance characteristics of the carbide are determined by the hardness, transverse rupture strength and fracture toughness. The surface appears clean and flat without any flaws or cracks whereas the epoxy-metal interface shows incomplete binding or bumps in the adhesive layer edge because of less adhesive curing and density variations. In this regard, the characteristic of robust adhesion is clearly revealed from the C-scan showing the homogeneity of the metal binder phase.

**Fig. 5:**
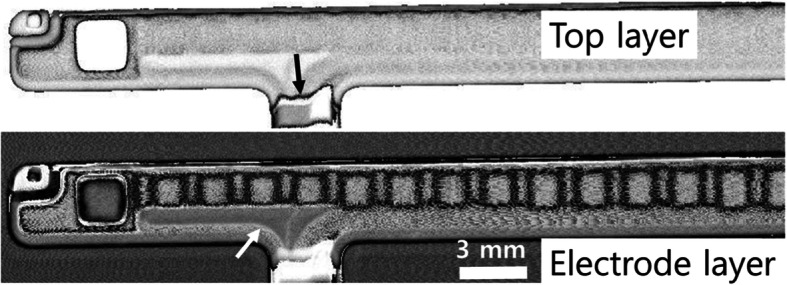
Image showing the flat panel bonding between a glass and the electrodes, obtained from a C-scan (50 MHz transducer, lateral resolution ~ 30 μm, 512 px × 128 px)

Figure 5 shows the internal non-destructive inspection of the flat panel bonding. Shadows of the electrode arrays and the irregular patterns of bonding (arrows) can be clearly seen at the electrode layer. The resin adhesion in the bottom row appears to overflow because of the inappropriate heating and curing conditions. Applying an appropriate amount of resin to a pad is very important for ensuring the improved optical performance and durability achieved by removing the airgaps (Bertocci et al. 2019). If the quantitity of resin applied is too small, it can result in empty spaces or holes inside. Good adhesion between the flat panel and the electrodes increases the robustness of the display (i.e., resistance to shock, vibration and moisture). Choosing a resin with an optimized refraction index is very crucial in the flat panel for a satisfactory display contrast and visibility. In addition, an adequate bond strength allows a conductive interconnection after the heat pressure process is performed for adhesive curing (Cruz et al. 2017; Yang et al. 2020). In the manufacturing process, the bonding of the display affects the total assembly time and the final display quality (Park and Lee, 2019).

**Fig. 6:**
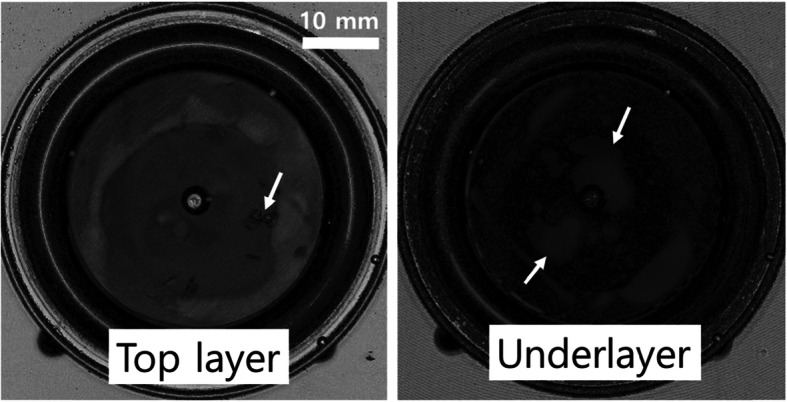
Image of the EPDM flange obtained from its C-scan (50 MHz transducer, lateral resolution ~ 30 μm, 512 px × 512 px)

The depth C-scan images of an Ethylene-Propylene-Diene Monomer (EPDM) rubber are presented in Fig. 6. The EPDM rubber is a widely used elastomer that is used for sealing and insulation and provides resistance to ultraviolet radiation, oxidation, aging, and moisture. It is an alternative rubber to silicone and nitrile owing to its efficient resistance to abrasion and tearing. It also provides high tensile strength and elasticity to provide high heat resistance and exhibits a – versatile temperature range from − 70 to 250 °C. In the figure, scratch and stains are observed in the left image at the top layer of the used rubber indicating deterioration at the surface. At the underlayer, inhomogeneous images are observed owing to delamination or elastic deformation which often occurs as a result of premature crosslinking or vulcanization at the contact layer (Wang and Yan, 2018).

**Fig. 7:**
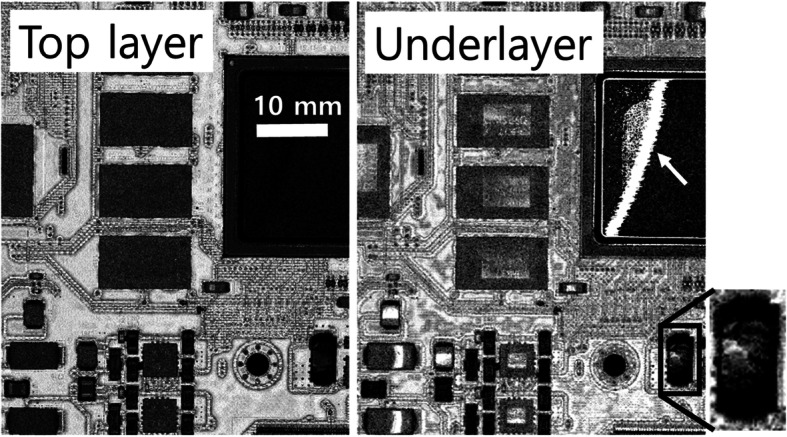
Image of a large area PCB (45 × 60 mm^2^) obtained from its C-scan (50 MHz transducer, lateral resolution ~ 30 μm, 480 px × 640 px)

Microelectronics within plastic packages can be inspected with a confocal resolution provided by SAM (Wüst and Rupitsch, 2018; Wang et al. 2019; Zhu et al. 2019; Shannon et al. 2019; Qiu and Zhang, 2017; Qiu et al. 2018). Figure 7 shows an image of a large area (45 × 60 mm^2^) printed circuit board (PCB) obtained from its C-scan and reveals the features of its discrete components, such as ICs, memory chips, resistors, capacitors and inductors. The high depth resolution (ca. 2 μm) allows us to resolve the top and inner metallic layers of the assembly. The depth resolution is determined by the gate width (2 ns) of the ultrasonic pulse and the sound velocity within the material (Liang et al. 2019; Song et al. 2013).

A plastic quad flat pack on the PCB was imaged from the top to the underlayer. The images of the underlayer on the right side in Fig. 7 reveals diagonal dark and white features whose boundaries are between the die surface and the mold compound. This suggests an extensive delamination at the die attach level. Gating a planar C-SAM image at the underlayer level shows that the delamination is extensive, where the layer in the substrate has gradually separated. The rectangular defect pattern at the bottom right indicates a popcorn crack resulting from the sudden expansion of trapped moisture.

**Fig. 8:**
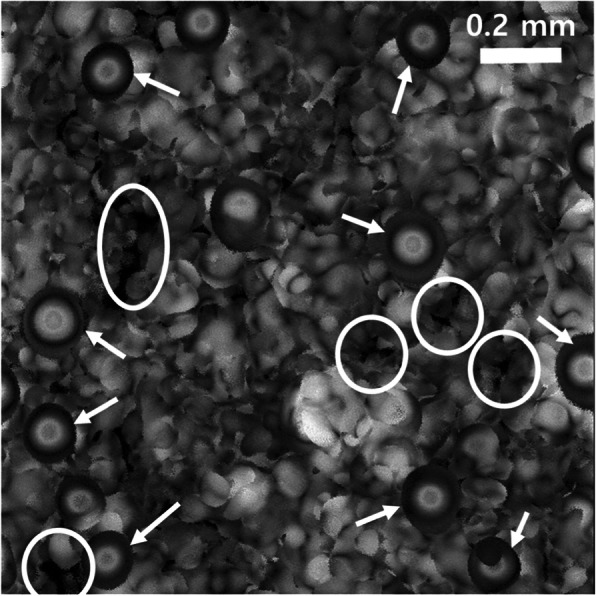
A lateral scan parallel to the scalp surface showing the microvessels (open circles) and hair follicles (arrows) (110 MHz transducer, lateral resolution ~ 15 μm, 1024 px × 1024 px)

A human subcutaneous tissue with 2 × 2 mm^2^ dimensions at ca. 400 μm depth of the scalp was scanned using SAM to probe the texture of the arrector pili muscle, sebaceous gland, lymph, microvessels (open circle) and hair follicles (arrows). The resultant image is shown in Fig. 8. The scalp hair follicles in Asian people are circular in shape with a diameter of 80–100 μm. The hair follicles clearly show a several layers of the internal root sheath - Henle’s layer, Huxley’s layer, and an internal cuticle that are continuous with the outermost layer of the hair fiber. It is important to understand and maintain scalp health since the scalp acts as a barrier to protect the cranial vault from physical trauma or infectious agents. High-contrast images of the subcutaneous tissue using SAM can help in the clinical diagnosis of hair thinning or loss treatment by analysing the sebaceous glands, microvessels, nerves, and follicles of the detailed layers of the scalp.

In recent years, advanced techniques such as ultrasound impedance microscopy, ultrasound speed microscopy, and scanning acoustic gigahertz microscopy have been developed for industrial and medical applications to provide additional information on the viscoelastic, anisotropic, internal stress, and nonlinear properties (Anastasiadis and Zinin, 2018; Demirkana et al. 2019; Hozumia et al. 2019). In ultrasound impedance microscopy, a plastic plate is inserted between the transducer and the biosample to be probed. The ultrasound propagates through the thin plastic plate which is considered as a reference signal, and is reflected at the interface between the plate and the sample. The reflected signal is compared with the reference signal and is regarded as the acoustic impedance (Saijo 2009). The impedance mode enables the analysis of the fine structures of the surface for assessing the biomechanics of the cells and the thinly sliced tissues.

Saijo et al. have investigated the acoustic properties of various organs and disease states by employing acoustic attenuation and sound speed images captured using a single pulsed wave instead of continuous waves used in the conventional SAM systems (Saijo et al. 2005). In ultrasound speed microscopy, the reflected waveforms from the glass substrate, which are considered as a reference signal, and tissue are obtained. The intensity and phase spectra are normalized with respect to the reference signal after applying a Fourier transform (Saijo et al., 2007a, b) and the information on the thickness, attenuation and sound speed is calculated (Saijo 2009; Saijo et al., 2007a, b). High-contrast images are acquired according to different elastic parameters on the basis of the distribution of the speed of sound.

SAM is becoming a diversified and favorite tool providing a unique microscopy modality for non-invasive inspection of industrial and medical samples in a short time duration and providing high-quality analyses. Novel acoustic techniques such as a relavant lens design and focus achieved using acoustic waves are highly prospective and motivating for many applications to adopt SAM as a versatile and quantitative evaluation tool.

## Conclusion

The basic concept, operation, and applications of acoustic microscopy for analyzing nontransparent objects have been described in this paper. A reliable non-destructive acoustic method provides an efficient inspection of defects, failure, delamination, and cracks at different depths. SAM is a useful technique for fast and convenient measurement of large area samples to probe their detailed internal structures layer by layer. A large variety of inspection modes such as vertical, horizontal, and diagonal cross-sections are possible employing the focus pathway and image reconstruction algorithm determined by the change in the acoustic impedance at the interface of material layers or defects. The use of these SAM is a unique imaging technique as it provides mechanical stress and high-resolution depth information that cannot be directly visualized by other techniques such as optical microscopy, X-ray computer-aided tomography, infrared thermography, magnetic resonance imaging and optical coherence tomography imaging modalities.

## Data Availability

The datasets used and/or analyzed during the current study are available from the corresponding author on reasonable request.

## Abbreviations

SAM: Scanning acoustic microscopy
AMI: Acoustic Micro Imaging
LiNbO_3_: Lithium niobate
PMN-PT: Lead magnesium niobate-lead titanate
TT-scan: Transmission through scan
EPDM: Ethylene-Propylene-Diene Monomer
PQFP: Plastic quad flat pack
PCB: Printed circuit board
